# Peter Doherty, Nobel Laureate: Questions and Reflections Concerning MHC Restriction and other Fruits of a Life of Biomedical Erudition

**DOI:** 10.20411/pai.v3i2.260

**Published:** 2018-12-21

**Authors:** Neil S. Greenspan

**Affiliations:** 1 Case Western Reserve University, Cleveland, Ohio

**Keywords:** T-cell recognition, Viral immunology, Vaccines, The nature of scientific progress

## INTRODUCTION

In 1974, Peter Doherty and Rolf Zinkernagel published a landmark article in *Nature* [1] that described the ability of lymphocytic choriomeningitis virus (LCMV)-specific cytotoxic T cells to lyse LCMV-infected, ^51^Cr-labeled target cells if the target cells shared class I major histocompatibility complex (MHC) molecules with these T cells. Surprisingly, infected and labeled target cells with disparate class I MHC molecules were not lysed. This phenomenon, which came to be known as “MHC restriction,” was a major advance in our understanding of the way in which T cells recognize antigen and was ultimately the basis for the awarding of the 1996 Nobel Prize in Physiology or Medicine to Peter Doherty and Rolf Zinkernagel. Readers interested in more information on Dr. Doherty or on MHC restriction are referred to the relevant pages of the Nobel Prize website [2].

### The Nature of the Transformation in Understanding of T-Cell Recognition Caused by the Discovery of MHC Restriction

**Neil Greenspan**

In your *Immunology Today* article [3] with Rolf in 1997, you refer to the discovery of MHC restriction as a “paradigm shift.” Had you read Thomas Kuhn's book, *The Structure of Scientific Revolutions* [4], where the term originated?

**Peter Doherty**

Yes, but a very long time ago.

**NG**

Kuhn distinguished between what he called “normal” science, which corresponded to experimental or observational work within a reigning paradigm, and “revolutionary” science, which corresponded to experimental or observational investigation after the operative conceptual scheme has been, in some sense, overturned. Do you believe that these categories accurately described research in cellular immunology before and after your seminal 1974 paper in *Nature* [1] demonstrating MHC restriction for mouse cytotoxic T cells (CTL) responding to lymphocytic choriomeningitis virus (LCMV)?

**PD**

Yes, the MHC gives it away … the “strong transplantation molecules were all about graft rejection.” We showed that they were about “surveillance of self.” Many found it very hard to get their heads around that switch.

**NG**

Although some individuals struggled with the new perspective on MHC molecules, the basic idea caught on relatively quickly and did not require, as was supposedly the case for quantum physics, the dying off of the more senior investigators. Would you agree?

**PD**

The basic phenomenon of MHC-restricted CTL activity was soon accepted, but the debate about what that meant continued. The 1-receptor/altered self model that we argued in our 1975 *Lancet* hypothesis provided an explanation for “Ir gene” effects and MHC polymorphism, but over the next decade, most prominent investigators (Harald von Boehmer, Hans Wigzell, Klaus Rajewsky, Charles Janeway, and ultimately Rolf Zinkernagel) seemed to favor the 2-receptor (1 for MHC, 1 for antigen) idea. As I recall, Janeway authored a meeting report in *Immunology Today* in the early 1980's that said, in effect, “Why are we still arguing about the T-cell receptor? It's encoded by an Ig heavy chain gene.” That position, which made no sense from what we knew about T-cell specificity, was only cleared up in the mid 1980's.

**NG**

Another major notion of Kuhn's was that the key terms in a given research paradigm were incommensurable with the key terms in a newer paradigm in the same field. Was that your experience in viral immunology in the years after 1974?

**PD**

The MHC should be called the SSC, the self-surveillance complex. There was no real paradigm for how CD8 T cells (in particular) work in virus infections.

**NG**

I think that Kuhn was claiming that the fundamental concepts in old and new paradigms, for example, atomic physics pre- and post-quantum mechanics, had different definitions making communication between adherents of these different frameworks difficult. My recollection of the years following the discovery of MHC restriction is that communication among investigators was not made especially problematic. However, it did take time for individuals in fields outside of immunology to understand MHC restriction and the implications of this phenomenon.

**PD**

The division within the field was very much into the “1-receptor” vs “2-receptor” camps. Outside the field many physicians, for example, have always (until the new immunotherapies) regarded immunology as intellectually difficult. And CD8+ T cells in the clinical mind (at least) were “suppressors.”

**Interpretation of Description of MHC Restriction of CTL in Nature Article**

**NG**

In your initial paper of 1974 [1], Rolf and you demonstrate that using 3 different inbred strains with distinct H-2 haplotypes, T cells are cytotoxic only to infected target cells that display surface class I molecules shared with those T cells. The simple interpretation is that CTL can only recognize viral antigen (in some form, later shown to be a peptide) in association (in some sense) with self MHC.

However, had you tested the CTL you generated in mice of 1 of the 3 H-2 haplotypes represented in your initial experiments against infected target cells from dozens of H-2 haplotypes and displaying in some cases class I molecules differing from self MHC class I molecules at only 1 or 2 amino acids, cytotoxicity against targets with nonself H-2 molecules would likely have been observed. Results of this sort have been obtained with human CTL and human target cells displaying the relevant nominal antigen. Are you receptive to the notion that MHC restriction is less than absolute, as it is typically presented?

**PD**

Nothing is absolute. At that time many HLA types (in particular) were very poorly defined. In any case, the point doesn't matter. The importance of the whole thing was in showing that anti-viral (or anti-cancer etc) CTLs are focused onto the cell surface via the MHC. The discrimination between K^b^ and K^bm1^ was a key finding.

**NG**

In the context of organ and hematopoietic cell transplants, the ability of self-MHC-restricted T cells to also respond to allogeneic MHC molecules is of high clinical significance. That such reactivity was not selected for over evolutionary history is worth noting, but in the medical arena the brute fact of such consequential cross-reactivity cannot be ignored.

**PD**

Sure, it's a medical problem, but is a solution likely to come from focusing on this issue? How would you take it forward, either conceptually or practically?

**NG**

Specifically, would you accept a slight re-statement of MHC restriction such as the following: T cells recognize nominal (eg, viral) antigen in association with self MHC molecules (of the appropriate class) and a subset of nonself molecules of the same class that are highly similar to self MHC molecules in primary structure? This perspective suggests that MHC restriction is ultimately best viewed as probabilistic because recognition by T-cell receptors (TCR), like antibodies, cannot be absolutely specific. Consequently, a single TCR may be able to recognize a particular peptide in association with a particular self MHC molecule and 1 or more MHC molecules with at least 1 difference in amino acid sequence from the corresponding self molecule.

**PD**

Sure, no problem. What's important is what the data shows. This is science, not religious dogma.

**NG**

What the data showed for Berenice Kindred and D.C. Shreffler (1972) [5] or David Katz and Baruj Benacerraf (as early as 1973) [6] was insufficient for them to make the inferences that Rolf and you made. Are you accepting of the view that the conceptual framework you and Rolf used in interpreting your data made a difference in what conclusions you drew?

**PD**

As I recall, I don't think Kindred and Shreffler concluded anything much and I know, for a fact, that (in 1975 at least) Don Shreffler (who was a good guy) thought our ideas were crazy. Katz and Benacerraf had come to some very premature conclusions regarding the “Ir gene products” (they thought they were the TCRs) that led them down the wrong path. César Milstein called them on this, but it was only when Benacerraf heard me talk in Boston, then saw our subsequent *Nature* and *Lancet* articles that he changed his mind. Katz stuck with their original idea.

We all work within a conceptual framework though, hopefully, we can have the insight to abandon it when it no longer has explanatory value. As experimentalists, the debate was around the “1-receptor/altered self” vs “2-receptor/physiologic recognition” idea. In 1 experiment, I thought we had some evidence for the latter (which was a problem), but it didn't hold up.

### Immunology and Philosophy of Science

**NG**

Do you believe that at least some of the questions under consideration by philosophers of science are relevant to active biomedical experimentalists?

**PD**

I haven't really read much philosophy of science, apart from early contact with Karl Popper and Kuhn. My contact with philosophers over the years does make me think that they can help to focus thinking processes. A philosopher friend on the local scene has an active consultancy that involves introducing business executives to philosophical principles.

**NG**

Picking up on your point about conceptual clarification, would you be amenable to the claim that conceptually minded immunologists have helped to clarify relevant ideas such as “epitope,” “specificity,” and “memory?”

**PD**

Not for me, but that likely reflects my limitations. Who were these “conceptual immunologists?” Mel Cohn was right to think in terms of 2 signals but otherwise was, I think, neither particularly helpful nor clear in his thinking.

The use of “epitope” is especially confused. My view is it should be the MHC-peptide (MHCp) complex, but many use it to refer to the peptide alone.

Specificity is being clarified by the structural biologists, but we also need to build avidity/affinity into that.

To my mind, Rolf got into an untenable position by confounding “memory” and “protection.” The whole effector memory T cells (TEM) central memory T cells (TCM) idea may have been useful for argument and for setting up as a “straw man” when writing papers, but it has often locked people into being too rigid in their thinking. Immunology has always provided a happy home for “splitters,” but the idea of rigidly defined lineages based on cell-surface “marker” expression is often over-interpreted, though whole careers can be built on it.

**NG**

What roles do you see for experiment, formal theory, and conceptualization in experimental immunology? Do you agree that your published studies on MHC restriction were more consequential than other studies that were published just before, roughly contemporaneously, or just after yours and generated similar data because the conceptual framework that Rolf and you formulated yielded more profound implications, such as potentially accounting for MHC allelic diversity?

**PD**

Our discovery was clearly accidental, and we managed to think that through to some useful conclusions**.** Part of the advantage we had over others working with, say, the MHC II molecules was that we had a better assay and, though the results in those early papers may not look all that spectacular now, the data were much clearer.

### Attributing Credit for Advances in Scientific Understanding

**NG**

In 1995, after you won the Lasker Basic Medical Research Award [7], I congratulated you and expressed the view that you and Rolf deserved the recognition associated with a Nobel Prize despite my concerns about the limitations of such awards. What are your thoughts about your contribution, with Rolf, to elucidating the nature of antigen recognition by T cells versus the contributions of those such as Kindred and Shreffler [5], Katz and Benacerraf [6], Michael Bevan [8], Gene Shearer [9], and Ethan Shevach and Alan Rosenthal? [10]

**PD**

Mike Bevan came later. Gene Shearer was closest to getting it right, I forget what Kindred and Shreffler did, and the others all misinterpreted. Katz and Benacerraf were arguing that the “Ir genes” were some sort of T-cell receptor. Milstein called them on this and pointed out that they had absolutely no evidence, but they still persisted with the argument.

**NG**

I appreciate your comments on the superior directness and therefore clarity of the cytotoxicity assay versus the assays used for the studies involving the sharing of class II MHC antigens (alleles) between T cells and B cells as a requirement for robust T-cell-dependent antibody production. In addition, perhaps because you were working with a virus, an actual pathogen versus haptens or other model antigens in the somewhat related studies by others, would you agree that your insight to connect your results to evolutionary arguments was critical in achieving the high impact of your findings?

**PD**

Yes. My thinking has been driven by thinking in terms of what Burnet described as “teleological Darwinism.” I was also helped by the fact that I'd not been heavily exposed to much of the then current thinking, and jargon, of immunology.

**NG**

Do you believe the Nobel Prizes in science have a net positive impact on science as currently operated, or do you believe that there are modifications that could be implemented, in principle, for these or other awards that would better reflect the complexity of the origins of discoveries and/or be ultimately better for the advancement of basic research?

**PD**

They provide some broad publicity for science and, in some cases, some outstanding spokesmen and activists, for example, Rich Roberts and Steven Chu. There are also a lot of other big science prizes, so it's not as though they are exclusive.

### Relevance of Evolutionary Ideas to the Impact of the Discovery of MHC Restriction

**NG**

The experiments performed by Rolf and you on MHC restriction are widely regarded as the most critical in establishing the concept. What role do you believe is played in that assessment by your hypothesis that MHC restriction could provide an evolutionary explanation for the extraordinary allelic diversity at class I (and class II MHC) loci?

**PD**

I don't know but, in my “lay” book *Their Fate is Our Fate* I highlight the evidence from the chicken (has only 1 MHC I locus) supporting that view.

**NG**

At the point when you put forth the hypothesis that MHC restriction could favor allelic heterozygosity at MHC class I loci how much had you studied evolutionary mechanisms?

**PD**

I've never really studied evolutionary mechanisms, but my science has been guided by what I'd refer to as “teleological Darwinism.”

**NG**

Can you briefly delineate what you mean by “teleological Darwinism?”

**PD**

If an interpretation makes sense in terms of evolution and selective advantage, there is some possibility that it may be useful.

**Reconciling MHC restriction and Potent T-Cell-Mediated Alloimmunity**

**NG**

The citation for Lasker Basic Medical Research Award [11] suggests that MHC restriction helped to explain the alloimmune response mounted by T cells against allogeneic tissues. However, one could argue that it is not obvious how to reconcile MHC restriction with alloimmunity. Did you think about this issue in the immediate aftermath of your discovery in 1974?

**PD**

I think you raised the issue of cross-reactivity above, which is where I lost interest in the question.

**NG**

Why does the phenomenon of cross-reactivity extinguish your interest?

**PD**

We thought about it a lot early on, then there were some demonstrations of “dual” (self+x and allo) reactivity (Michael J. Bevan, Thomas J. Braciale). After that, I lost interest for a time, though I think useful insights will come from the structural biologists as they press on with analyzing TCR/MHCp “microanatomy.”

**NG**

Do you currently have a preferred means for accounting for MHC restriction and the relatively high potency of T-cell responses to alloantigens?

**PD**

Expect that we will get better insights from systematic TCR/MHCp co-crystal analysis using mutated MHCs and substituted peptides. I just think of it as the “micro-anatomy of surface-surface interactions.”

### Viral Immunology

**NG**

Do you believe there are a small number of specific principles applicable to viral immunology that can be concisely delineated?

**PD**

Apart from the obvious facts regarding TCR and Ig recognition, the roles of cytokines, chemokines, etc make this very complex.

**NG**

What are your views on the relative importance of circulating virus-specific antibody versus virus-specific memory B cells in vaccine-induced immunity to viral pathogens for which we vaccinate?

**PD**

Both are important.

**NG**

Do you regard long-lived plasma cells and the antibodies they produce as part of immunological memory?

**PD**

Yes.

**NG**

Can you offer any generalizations or ways to think about the relative roles of humoral versus T-cell-mediated in immunity to viruses?

**PD**

Vaccines generally work by antibody. Depending on the virus, CD8+ T cells can be more (LCM) or less (herpes) important in initial virus control, and a more rapid recall response can help terminate the infection earlier. Maybe there's a role for a cross-reactive “belt and braces” (IG + T cell memory) flu vaccine. Not sure about resident T memory cells … are they “memory” if there's persistent, low level infection? Much of the initial control of herpes infections, for example, is by CD4+ T cells secreting gamma interferon.

**NG**

Do you care to offer any thoughts about the likelihood that biomedical scientists will ultimately be successful in producing a successful vaccine for human immunodeficiency virus (HIV)?

**PD**

We can hope, but I can't see how this can work. The “cross-reactive Ig epitope” approach does not seem to have delivered. Problem is that any HIV vaccine has to deliver “sterilizing immunity.”

**NG**

Are you anticipating eventual success in formulating a universal influenza virus vaccine?

**PD**

More likely than with HIV. A flu vaccine only has to deliver partial protection that shortens the course of the infection and diminishes the degree of damage.

**NG**

What are your thoughts about the eventual success of efforts to exploit immunological cells and molecules to implement a cure of HIV infection by eliminating the latent reservoir of infected cells?

**PD**

The problem here is to flush out all the cells carrying HIV DNA. Hard to see how that will be possible.

**NG**

In light of the relatively recent development of highly effective chemotherapeutic agents for hepatitis C virus (HCV), do you believe it is still sensible to invest the funds necessary to pursue a vaccine for HCV?

**PD**

I would not make it a priority.

## References

[1] Zinkernagel RM, Doherty PC Restriction of in vitro T cell-mediated cytotoxicity in lymphocytic choriomeningitis within a syngeneic or semiallogeneic system. Nature. 1974;248(5450):701–2. PubMed PMID: 4133807. 4133807 10.1038/248701a0

[2] Doherty PC Peter C. Doherty - Biographical NobelPrize.org: Nobel Media 1996 [accessed on 11/26/18] Available from: https://www.nobelprize.org/prizes/medicine/1996/doherty/biographical/.

[3] Zinkernagel RM, Doherty PC The discovery of MHC restriction. Immunol Today. 1997;18(1):14–7. PubMed PMID: 9018968. 9018968 10.1016/s0167-5699(97)80008-4

[4] Kuhn TS The Structure of Scientific Revolutions 2nd ed. Chicago and London: University of Chicago Press; 1962.

[5] Kindred B, Shreffler DC H-2 dependence of co-operation between T and B cells in vivo. J Immunol. 1972;109(5):940–3. PubMed PMID: 4538719. 4538719

[6] Katz DH, Hamaoka T, Dorf ME, Benacerraf B Cell interactions between histoincompatible T and B lymphocytes. The H-2 gene complex determines successful physiologic lymphocyte interactions. Proc Natl Acad Sci U S A. 1973;70(9):2624–8. PubMed PMID: 4126264. Pubmed Central PMCID: 427069. 4126264 10.1073/pnas.70.9.2624 PMC427069

[7] 1995 Lasker Awards: Albert And Mary Lasker Foundation; [accessed on 11/26/18] Available from: http://www.laskerfoundation.org/awards/year/1995/. 10.1001/jama.2014.11986 26372599

[8] Bevan MJ The major histocompatibility complex determines susceptibility to cytotoxic T cells directed against minor histocompatibility antigens. J Exp Med. 1975;142(6):1349–64. PubMed PMID: 53263. Pubmed Central PMCID: 2190072. 53263 10.1084/jem.142.6.1349 PMC2190072

[9] Schmitt-Verhulst AM, Shearer GM Bifunctional major histocompatibility-linked genetic regulation of cell-mediated lympholysis to trinitrophenyl-modified autologous lymphocytes. J Exp Med. 1975;142(4):914–27. PubMed PMID: 52685. Pubmed Central PMCID: 2189948. 52685 10.1084/jem.142.4.914 PMC2189948

[10] Rosenthal AS, Shevach EM Function of macrophages in antigen recognition by guinea pig T lymphocytes. I. Requirement for histocompatible macrophages and lymphocytes. J Exp Med. 1973;138(5):1194–212. PubMed PMID: 4542806. Pubmed Central PMCID: 2139433. 4542806 10.1084/jem.138.5.1194 PMC2139433

[11] 1995 LASKER AWARDS: 1995 Albert Lasker Basic Medical Research Award: Albert And Mary Lasker Foundation; [Accessed on 11/26/18]. Available from: http://www.laskerfoundation.org/awards/show/t-cells-and-immune-defense/.

